# Inverse Association of Plasma Molybdenum with Metabolic Syndrome in a Chinese Adult Population: A Case-Control Study

**DOI:** 10.3390/nu13124544

**Published:** 2021-12-18

**Authors:** Ben Li, Yue Huang, Cheng Luo, Xiaolin Peng, Yang Jiao, Li Zhou, Jiawei Yin, Liegang Liu

**Affiliations:** 1 Department of Nutrition and Food Hygiene, Hubei Key Laboratory of Food Nutrition and Safety, School of Public Health, Tongji Medical College, Huazhong University of Science and Technology, Wuhan 430030, China; liben1219@163.com (B.L.); d201881303@hust.edu.cn (Y.H.); 2017506049@hust.edu.cn (C.L.); xiaolinpeng@hotmail.com (X.P.); zhouli2020@wust.edu.cn (L.Z.); 2 Ministry of Education Key Lab of Environment and Health, School of Public Health, Tongji Medical College, Huazhong University of Science and Technology, Wuhan 430030, China; d202181662@hust.cn; 3 Department of Non-Communicable Disease Prevention and Control, Shenzhen Nanshan Center for Chronic Disease Control, Shenzhen 518000, China

**Keywords:** molybdenum, metabolic syndrome, dose-response relationship, case-control study

## Abstract

Molybdenum has been found to be associated with metabolic disorders. However, the relationship between molybdenum and metabolic syndrome (MetS) is still unclear. A large case-control study was conducted in a Chinese population from the baseline of Ezhou-Shenzhen cohort. A total of 5356 subjects were included with 2678 MetS and 2678 controls matched by sex and age (±2 years). Medians (IQRs) of plasma molybdenum concentrations were 1.24 μg/L for MetS cases and 1.46 μg/L for controls. After adjustment for multiple covariates, the odds ratio (OR) and 95% confidence intervals (CIs) for MetS were 1.00 (reference), 0.71 (0.59–0.84), 0.56 (0.46–0.68), and 0.47 (0.39–0.58) across quartiles of plasma molybdenum, and per SD increment of log-transformed molybdenum was associated with a 23% lower risk of MetS. In the spline analysis, the risk of MetS and its components decreased steeply with increasing molybdenum and followed by a plateau when the cutoff point was observed around 2.0 μg/L. The dose-dependent relationship of molybdenum with MetS remained consistent when considering other essential elements in the Bayesian kernel machine regression (BKMR) model. In our study, higher plasma molybdenum was significantly associated with a lower risk of MetS, as well as its components, in a dose-response manner.

## 1. Introduction

The metabolic syndrome (MetS) is a constellation of metabolic abnormalities including abdominal obesity, dyslipidemia, hypertension, and disturbed glucose and insulin metabolism [1], all of which were well documented high-risk factors for both cardiovascular diseases (CVD) and type 2 diabetes mellitus [2,3,4]. The prevalence of MetS has increased strikingly over the past few decades and is now at epidemic proportions worldwide [5,6,7]. A recent report indicated that one-third of Chinese adults had MetS due to the rapid socio-economic growth within a short time [8]. Growing evidence suggested that essential elements might impact the onset and progression of MetS [9,10,11,12].

Molybdenum (Mo) is an essential trace element for the normal human physiology and functions as a cofactor for several enzymes in metabolism, including xanthine oxidoreductase (XOR), aldehyde oxidase (AO), sulfite oxidase (SO), nitrite reductases, and mitochondrial amidoxime reducing component (mARC) [13,14,15,16,17]. Among general adults, legumes, grain products, nuts, and dark-leafy vegetables, which showed beneficial effects for human metabolic health, are the major food sources of molybdenum [17]. Evidence from in vitro and animal studies has demonstrated that molybdenum as an antioxidant and activator of several antioxidant enzymes could alleviate lipid peroxidation, downregulate triglyceride and phospholipid species, enhance glucose-induced insulin secretion, and mitigate elevated blood pressure [18,19,20,21,22]. However, epidemiological evidence regarding molybdenum and metabolic disorders are conflicting. Some researches revealed circulating molybdenum was associated with a lower risk of diabetes, and hypertension [23,24,25,26], whereas others found that higher molybdenum was positively associated with hyperglycemia, hyperlipidemia, and hypertension [27,28,29]. The evidence of molybdenum and MetS is limited in humans [30], and their dose-response association is still unclear.

To address the knowledge gap as mentioned above, we performed a large case-control study to examine the association between plasma molybdenum levels with the risk of MetS and its components in a Chinese adult population.

## 2. Materials and Methods

### 2.1. Study Population

The study was conducted based on Ezhou-Shenzhen cohort (EZSZ) baseline data. The EZSZ was initiated to investigate the impacts of nutrition, lifestyle, and genetic factors with chronic diseases in Ezhou and Shenzhen city (in China). Ezhou cohort had been described in our previous report [31]. Briefly, between 2013 and 2015 in Ezhou and between 2017 and 2020 in Shenzhen, 9328 adult residents were enrolled for a baseline investigation. All participants included at baseline received medical examination in two survey centers (5533 participants at Echeng Steel hospital or Ezhou Center of Diseases Control and Prevention [Ezhou, China]; 3795 participants at Community Health Service Center of Nanshan District [Shenzhen, China]). A total sample of 7787 participants completed semi-structured questionnaires, received physical examinations, and provided fasting blood specimens. In the present study, individuals meeting any of the following conditions were further excluded: age < 18 years, age > 80 years, BMI ≥ 40 kg/m^2^, any clinically significant neurological, endocrinological or other systemic diseases, any acute illness, and chronic inflammatory or infective diseases, as well as those with missing plasma molybdenum concentrations. Finally, 2678 MetS cases and 2678 controls were included in the current analysis, which were 1:1 matched on sex and age (±2 years). Written informed consent was obtained from each subject enrolled and all the subjects were of Chinese Han ethnicity. This research protocol was approved by the Ethics and Human Subject Committee of Tongji Medical College, Huazhong University of Science and Technology.

### 2.2. Definition of MetS

The metabolic syndrome was defined according to the diagnostic criteria proposed by the Joint Interim Statement [4]. Participants defined as MetS had to meet at least three of the following criteria: (1) abdominal obesity: waist circumference ≥90 cm in men or ≥80 cm in women (following Asian population categorical cut points); (2) hypertriglyceridemia: triglyceride (TG) ≥ 1.7 mmol/L (150 mg/dL); (3) low levels of high-density lipoprotein cholesterol (HDL-C): HDL-C < 1.0 mmol/L (40 mg/dL) in men or <1.3 mmol/L (50 mg/dL) in women; (4) hypertension: systolic and diastolic blood pressure ≥ 130/85 mmHg and/or use of antihypertensive medication; (5) hyperglycemia: fasting plasma glucose (FPG) ≥ 5.6 mmol/L (100 mg/dL) and/or current use of anti-diabetic medication and/or self-reported history of diabetes.

### 2.3. Data Collection

Demographic, behavioral, and medical information were collected from questionnaires through face-to-face interviews. Personal information consisted of sex, age, smoking status (classified as current, former, and never), drinking status (classified as current, former, and never), physical activity (classified as at least 1 h per week for more than half a year or no), educational level (classified as none or elementary school, middle school, and high school or college), family history of diabetes (yes or no), and history of the disease (diabetes, hypertension, and hyperlipidemia). Anthropometric measurements including height (m), weight (kg), waist and hip circumference, and blood pressure, which were measured with standardized techniques by trained and certified technicians. Waist circumference was measured at the midpoint between the lower rib and iliac crest in a standing position, and at the end of expiration by using a measuring tape to the nearest 0.1 cm. Hip circumference was measured as the greatest gluteal circumference. Blood pressure was obtained in a seated position after 5 min of seated rest. Body mass index (BMI, kg/m^2^) was calculated as weight in kilograms divided by the square of height in meters. The waist-to-hip ratio (WHR) was also used as an index of fat distribution.

### 2.4. Laboratory Measurements

After overnight fasting (10–12 h), venous blood samples were collected in ethylene diamine tetraacetic acid anticoagulative vessels by the clinic nurse. Clinical parameters, such as fasting plasma glucose (FPG), total cholesterol (TC), triglyceride (TG), high-density lipoprotein cholesterol (HDL-C), low-density lipoprotein cholesterol (LDL-C), were further determined with standardized kits. The plasma was separated and stored at −80 °C for subsequent analysis of molybdenum and other essential elements.

### 2.5. Measurement of Plasma Essential Elements Concentrations

Plasma molybdenum and other essential elements concentrations were detected by inductively coupled plasma mass spectrometry (Agilent 7700 Series ICP-MS; Tokyo, Japan) in the Ministry of Education Key Laboratory of Environment and Health at Tongji Medical College of Huazhong University of Science and Technology, as described in our previous study [32]. In the daily measurement, specimens from MetS cases and control subjects were randomly assayed. The detection limit of molybdenum was 0.0036 μg/L, and the concentration of the lowest standard solution (0.02 μg/L) was considered as the limit of quantitation. For quality assurance, the certified reference materials ClinChek No. 8885 (level I and level II) human plasma controls for metals were analyzed in every 20 samples. The concentrations of molybdenum were 2.20 ± 0.17 μg/L (certified: 1.91 ± 0.57 μg/L) and 6.68 ± 0.35 μg/L (certified: 6.58 ± 1.31 μg/L) for level I and level II, respectively. The intra-assay and inter-assay coefficients of variation of plasma molybdenum were both <5% and all participants had plasma levels of elements above the detection limit. Recovery ranged from 80% to 120% and the relative standard deviation of the measured triplicate samples was always within 10% in the sample measurement process to ensure the accuracy and precision of the experimental methods.

### 2.6. Statistical Analysis

Continuous variables were expressed as mean (standard deviations) for normally distributed data and median (interquartile range) for non-normally distributed data, and categorical data were expressed as numbers (percentages). Student’s *t*-test and Mann-Whitney’s U test were performed to examine the significance of differences between MetS and control groups for continuous variables, and chi-square test for categorical variables. Plasma molybdenum concentrations were categorized into quartiles: Q1: ≤1.05 μg/L; Q2: 1.05–1.46 μg/L; Q3: 1.46–1.97 μg/L, and Q4: ≥1.97 μg/L. Conditional logistic regression analysis were conducted to calculate the ORs (95% CIs) by using the first quartile as the reference category. Tests of linear trend across increasing quartiles of molybdenum were performed by treating the median value of each quartile as a continuous variable in the logistic regression models. The potential confounders were stepwise adjusted in three models: Model 1 adjusted for sex, age (≤40, 40–49, 50–59, ≥60), and BMI (≤18, 18–23.9, 24–27.9, ≥28). Model 2 additionally adjusted for smoking and drinking status, physical activity, an educational level, family history of diabetes, and survey center (Ezhou and Shenzhen) based on model 1. In addition to the above traditional risk factors, we further adjusted for other essential elements in model 3, including plasma calcium, magnesium, iron, copper, chromium, selenium, and cobalt. BMI was entered as a covariate in all models, except for in models of abdominal obesity to avoid over adjustment. In addition, binary logistic regression model was conducted in stratified analyses to evaluate the consistency of the association between molybdenum and MetS according to participant characteristics in fully adjusted models. The interactions between these stratification variables and molybdenum were tested by adding multiplicative terms into the logistic regression models, with adjusting confounding variables mentioned above.

Restricted cubic spline (RCS) analysis was also performed to detect the shape of dose-response relationships of log-transformed molybdenum with the risk of MetS and its components with four knots at the 5th, 35th, 65th, and 95th percentiles of its distribution. The reference value was set at the 5th percentile, and the values outside the 2th and 98th percentiles were excluded.

Lastly, we further built the Bayesian kernel machine regression (BKMR) model (10,000 iterations for plasma elements data) to estimate the joint effects of molybdenum and other essential elements with the risk of MetS. The overall impact of the combined essential elements mixture on MetS in comparison to its 50th percentile was summarized. The difference of MetS risk for a change in element concentration between the 25th and 75th percentile was also displayed for each element when the other elements of the mixture were fixed at either the 25th, 50th, or 75th percentile levels. All elements were log-transformed for BKMR analysis to reduce skewness.

All statistical analysis was performed with SPSS 24.0 (SPSS Inc., Chicago, IL, USA), SAS 9.4 (SAS Institute Inc., Cary, NC, USA), and R software (version 4.0.2, R Foundation for Statistical Computing, Vienna, Austria). Statistical significance was inferred at a two-tailed *p* < 0.05.

## 3. Results

### 3.1. Characteristics of the Participants

Table 1 summarized the demographic, anthropometric, clinical, and behavioral information of 5356 participants (2678 MetS cases and 2678 controls). The mean age of the MetS cases and control subjects were 54.5 ± 10.9 and 54.8 ± 10.8 years, respectively, and 55.2% of the participants were men. Individuals with MetS were more likely to have lower plasma molybdenum concentrations (*p* < 0.01) and a higher prevalence of family history of diabetes (*p* < 0.01). As expected, higher levels of BMI, waist circumference, hip circumference, waist-to-hip circumference ratio, systolic blood pressure (SBP), diastolic blood pressure (DBP), fasting plasma glucose, triglycerides, and lower levels of HDL-C in the MetS group were observed compared with controls (*p* < 0.01 for all). In contrast, there was no significant difference observed in sex, age, total cholesterol, LDL-C, smoking and drinking status, physical activity, and an educational level between MetS and control groups.

### 3.2. Associations of Molybdenum with MetS and Its Components

The relations of plasma molybdenum with MetS and its components were presented in Table 2. The ORs (95% CIs) of MetS from the lowest to the highest quartiles of plasma molybdenum were 1.00 (reference), 0.71 (0.59–0.84), 0.56 (0.46–0.68), and 0.47 (0.39–0.58) after multiple adjusted for confounding factors (*p* for trend < 0.001). In addition, the OR (95% CIs) of MetS for each SD increment of log-transformed plasma molybdenum was 0.77 (0.71–0.83). Furthermore, similar inverse associations were found in the components of MetS. When compared the fourth quartile with the reference of molybdenum concentration, the ORs (95% CIs) were 0.53 (0.43–0.64), 0.64 (0.53–0.76), 0.68 (0.56–0.82), and 0.71 (0.58–0.86) for abdominal obesity, hypertriglyceridemia, low HDL-C and hypertension, respectively (*p* for trend < 0.01 for all). The results of stratified analyses by sex, age, BMI, current smoking and drinking status, physical activity, family history of diabetes, and survey center were shown in Table 3. The inverse relationship between plasma molybdenum and MetS was coincident in all subgroups. Significant differences in the sex-, BMI-, and drinking-specific subgroups were observed (*p* for interaction < 0.05). The inverse relationships seemed stronger among males, normal-weight individuals, and current drinkers.

### 3.3. Restricted Cubic Spline Analysis

**Figure 1:**
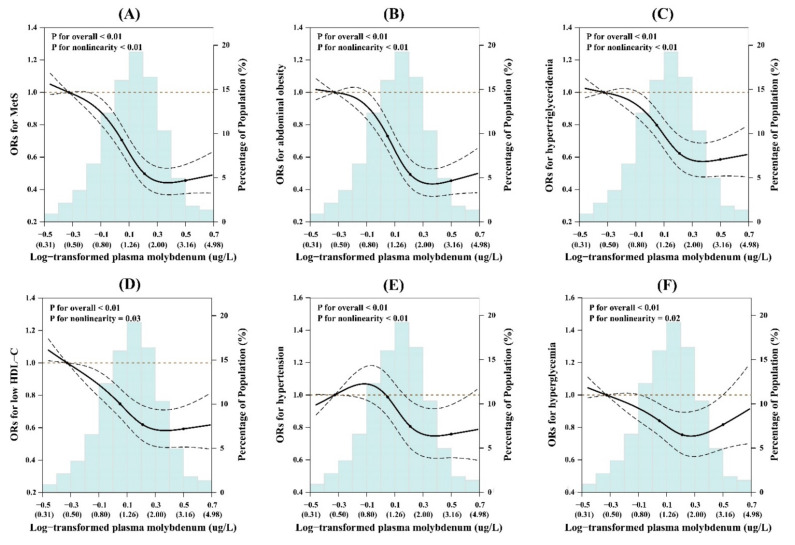
The restricted cubic spline for the associations of log-transformed plasma molybdenum with MetS and its components. (**A**) MetS; (**B**) abdominal obesity; (**C**) hypertriglyceridemia; (**D**) low HDL-C; (**E**) hypertension; (**F**) hyperglycemia. Knots were placed at the 5th, 35th, 65th, and 95th percentiles of the plasma molybdenum distribution. Results were adjusted for sex, age (≤40, 40–49, 50–59, ≥60), body mass index (≤18, 18–23.9, 24–27.9, ≥28), smoking (current, former, and never), drinking (current, former, and never), physical activity (yes or no), an education level (none or elementary school, middle school, and high school or college), family history of diabetes (yes or no), survey center (Ezhou and Shenzhen), and multiple essential elements (log-transformed plasma calcium, magnesium, iron, copper, chromium, selenium, and cobalt).

In the spline regression models, the risk of MetS and its components decreased considerably and then leveled off with increasing log-transformed molybdenum. The cutoff point was observed at plasma molybdenum concentration of around 2.0 μg/L (Figure 1). Plasma molybdenum levels showed non-linear relationships with risk of MetS (Figure 1A), as well as abdominal obesity (Figure 1B), hypertriglyceridemia (Figure 1C), low HDL-C (Figure 1D), hypertension (Figure 1E), and hyperglycemia (Figure 1F) (*p* for nonlinearity < 0.05 for all).

### 3.4. BKMR Analysis

**Figure 2:**
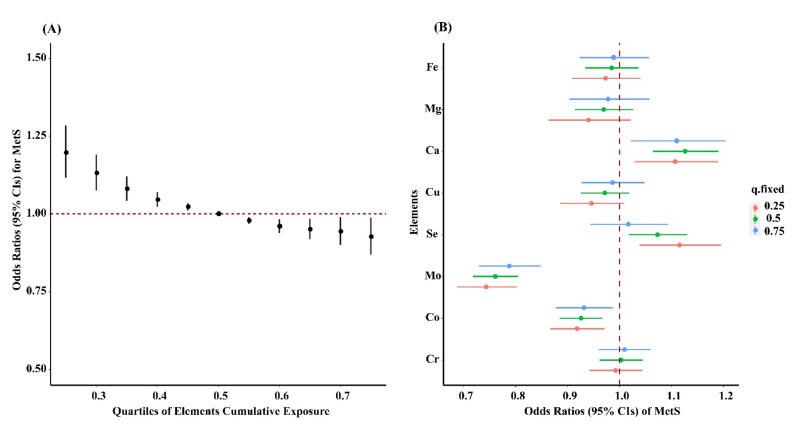
Joint effect of the essential elements mixture (calcium, magnesium, iron, copper, chromium, selenium, molybdenum, and cobalt) on MetS by using Bayesian kernel machine regression (BKMR) model. (**A**) Overall effect of the essential elements mixture estimates and 95% confidence intervals. (**B**) Single element association (estimate and 95% confidence intervals); this plot compares ORs of MetS when a single element was at the 75th vs. 25th percentile when all the other elements were fixed at either the 25th (red line), 50th (green line), or 75th percentile (blue line). Results were adjusted for sex, age (≤40, 40–49, 50–59, ≥60), body mass index (≤18, 18–23.9, 24–27.9, ≥28), smoking (current, former, and never), drinking (current, former, and never), physical activity (yes or no), an education level (none or elementary school, middle school, and high school or college), family history of diabetes (yes or no), and survey center (Ezhou and Shenzhen).

For eliminating the collinearity of other essential elements with molybdenum, we further used the BKMR model to confirm the results from regression models. When all elements were above their 50th percentile, the mixture of elements showed a significantly negative association with MetS prevalence, while a significant positive association was observed when all element concentrations were all below their 50th percentile, as compared to that when all elements were at the 50th percentile (Figure 2A). In addition, the inverse association between plasma molybdenum and MetS remained significant in a dose-response manner when each of the other essential elements was set at the corresponding median concentrations (Figure 2B).

## 4. Discussion

In this case-control study, we firstly revealed that plasma molybdenum was inversely associated with the risk of MetS and its components, independent of the established risk factors for MetS. In addition, the associations were robust in the stratified analyses. Furthermore, the dose-dependent relationship of molybdenum with MetS remained consistent when considering other essential elements in the BKMR model.

Daily molybdenum requirement has been set to 100–300 μg/d for adults by the World Health Organization (WHO), while the US established recommended daily allowance (RDA) of 45 μg/d for both men and women [33,34]. Estimates of molybdenum intake among different countries varied ranging from 123 to 275 μg/d [35,36]. However, it is challenging in free-living populations to accurately measure food and nutrient intakes, especially for molybdenum intake due to the limitations of food composition data. Inaccurate identification of molybdenum intake could lead to measurement errors and confuse the health effect of molybdenum. Plasma molybdenum were widely used as additional estimates of dietary nutrient intake. In our study, the medians of plasma molybdenum concentrations in the MetS group and control group were 1.24 (0.85–1.70) μg/L and 1.46 (1.05–1.97) μg/L, respectively, which was similar to previous reports [24,37]. The risk of MetS and its components decreased significantly with increasing molybdenum and followed by a plateau. The cutoff point was observed around the plasma molybdenum concentration of 2.0 μg/L. At present, there is no internationally acceptable value or range for plasma molybdenum concentration in the general population for chronic disease prevention. Our results need to be validated in other population and may provide an implication for establishing regulatory guidance range of plasma molybdenum concentration in the general population to manage and control MetS.

In the available literature, there is limited epidemiological evidence on the relationship between molybdenum and the risk of MetS. Only one cross-sectional study found no significant association of urinary molybdenum with MetS in a general Chinese population [30], which was not in line with our study. Although both plasma and urinary molybdenum are widely used as biomarkers of dietary molybdenum intake [38,39], the molybdenum concentrations were largely variable in urine, which might partly explain the discrepancies with our results. Besides, other possible reasons may also account for the inconsistencies, including different study designs, sample sizes, and adjusted covariates, etc. For instance, the current study applied a more rigorous case-control design with a larger sample size to provide sufficient statistical power. Meanwhile, other essential elements, which may interact with molybdenum, were considered to minimize the confounding effects. In our subgroup analyses, a cross region validation for the subpopulations living in Ezhou and Shenzhen was conducted to examine the robustness of our findings, which remained rather consistent results. Our findings also revealed that plasma molybdenum was reversely associated with blood glucose and triglyceride levels, which was supported by two prospective studies indicating that plasma molybdenum has a significant benefit in lowering glucose levels and homeostatic model assessments for insulin resistance (HOMA-IR) [24,25]. Consistent with our findings, two recent studies also found inverse associations of serum and urinary molybdenum with blood pressure [26,40]. However, contradictory relationships of urinary molybdenum with diabetes and HOMA-IR, as well as blood pressure, were also reported [27,28,29]. Additionally, the associations between molybdenum and other components of MetS have been less studied. A few studies suggested insignificant associations of molybdenum with waist circumstance and body mass index [41,42]. Further investigations are warranted to validate our results and explain the causality of molybdenum and MetS.

In the present study, significant interactions in the sex-, BMI-, and drinking-specific subgroup analyses were observed. In our study population, 99.14% proportion of individuals in the BMI ≤ 24 subgroup had normal weight (18 ≤ BMI ≤ 24), and these subjects with higher levels of plasma molybdenum were at sharply decreased risk of MetS. However, for those with BMI ≥ 24, the association between plasma molybdenum and MetS was attenuated. This suggested that susceptibility to the protective effects of molybdenum on MetS might vary substantially with ranges of BMI. Similar attenuated associations were also observed in females and nondrinkers. These findings should be cautiously interpreted, and further investigations are warranted to characterize the potential mechanisms behind it.

There is biological plausibility for the role of molybdenum in the development of MetS. First, potential activities of molybdate as an antioxidant and activator of other antioxidant enzymes have been demonstrated alleviating lipid peroxidation and lipid accumulation in diabetic rats [19,43,44]. Second, molybdenum-containing enzymes, as nitrite reductases, have been shown to convert nitrite from endogenous or dietary sources into nitric oxide (NO), which could increase cyclic guanosine monophosphate (cGMP) levels in target cells and then downregulate the contractile apparatus in vascular smooth muscle, thereby leading to a lowering of blood pressure [21,45,46]. Third, molybdate, as a phosphotyrosine phosphatase (PTPase) inhibitor, can mimic certain insulin actions to normalize blood glucose levels by increasing phosphorylation and autophosphorylation of both insulin receptor tyrosine kinase (insRTK) and cytosolic protein tyrosine kinase (cytPTK) substrate, which is beneficial to upregulate insulin signal transduction [47,48,49]. Glucose-lowering effects of molybdate were also observed in pancreatic beta cells through increasing cellular insulin content and enhancing both basal and glucose-induced insulin secretion [50,51].

There are several strengths of our study. First, this was the first study to evaluate the dose-response relationship of plasma molybdenum with MetS and its components in a large case-control study, which provided sufficiently statistical power and the wide range of molybdenum levels. Moreover, we measured molybdenum and other essential elements concentrations in individual plasma specimens, and examine the multiple-elements joint effect, which could provide more reliable estimates. However, this study also had several limitations. First, the case-control design could not establish any causal relationships of molybdenum with MetS and its components, and possible reverse causation cannot be fully excluded. Second, we lacked the information of dietary molybdenum consumption to explore the relation between molybdenum intake and MetS. However, bias of dietary recall may lead to measurement errors, and plasma molybdenum has been suggested to be a reliable biomarker to reflect molybdenum intake in individuals [38,39]. Third, although we controlled for multiple MetS risk factors including lifestyle habits and most of the other essential elements, we lacked the information on plasma zinc concentrations. Moreover, renal function might play a role in the association of molybdenum with MetS, but we do not have the data of estimated glomerular filtration rate (eGFR), therefore the possibility of residual confounding could not be ruled out. Finally, all participants in this study were of Chinese Han ethnicity, which could minimize the confounding effects by ethnic background but may limit the generalizability of the results to other ethnic groups.

## 5. Conclusions

In conclusion, we found that higher plasma molybdenum was significantly associated with a lower risk of MetS, as well as abdominal obesity, hypertriglyceridemia, low HDL-C, hypertension, and hyperglycemia in a dose-response manner. Our findings suggested that the low molybdenum status may contribute to, or be a consequence of, MetS, which could help us understanding the adverse effects of molybdenum deficiency in MetS development. However, limited relevant epidemiological and clinical researches make our results hard to interpret for clinicians, and further studies are anticipated to validate our results and elucidate the impacts of molybdenum on the pathogenesis and prognosis of MetS.

## Figures and Tables

**Table 1:** Characteristics of study participants.

Characteristics	MetS (*n* = 2678)	Controls (*n* = 2678)	*p* Value
Age (y)	54.5 (10.9)	54.8 (10.8)	0.286
Male, *n* (%)	1478 (55.2)	1478 (55.2)	1.000
BMI (kg/m^2^)	25.88 (2.99)	24.36 (2.78)	<0.001
Waist circumference (cm)	90.33 (7.84)	84.60 (8.19)	<0.001
Hip circumference (cm)	98.26 (6.31)	95.19 (6.28)	<0.001
Waist/hip ratio	0.92 (0.05)	0.89 (0.06)	<0.001
SBP (mmHg)	137.78 (20.14)	127.69 (19.85)	<0.001
DBP (mmHg)	82.80 (11.70)	76.96 (10.98)	<0.001
Fasting plasma glucose (mmol/L)	5.76 (5.23–6.30)	5.15 (4.80–5.51)	<0.001
Triglycerides (mmol/L)	1.89 (1.41–2.55)	1.15 (0.88–1.48)	<0.001
Total cholesterol (mmol/L)	4.99 (4.36–5.71)	4.94 (4.37–5.59)	0.137
HDL cholesterol (mmol/L)	1.20 (0.33)	1.43 (0.36)	<0.001
LDL cholesterol (mmol/L)	2.95 (0.94)	2.99 (0.90)	0.127
Smoking, *n* (%) ^a^			0.750
Current	622 (23.3)	602 (22.6)	
Former	277 (10.4)	270 (7.8)	
Never	1772 (66.3)	1796 (67.3)	
Drinking, *n* (%) ^b^			0.691
Current	692 (26.0)	714 (26.8)	
Former	198 (7.4)	205 (7.7)	
Never	1775 (66.6)	1744 (65.5)	
Physical activity, *n* (%) ^c^			0.111
Yes	1469 (55.3)	1521 (57.4)	
No	1189 (44.7)	1127 (42.6)	
Family history of diabetes, *n* (%) ^d^			<0.001
Yes	335 (12.7)	256 (9.7)	
No	2310 (87.3)	2390 (90.3)	
Educational levels, *n* (%) ^e^			0.414
None or elementary school	611 (23.0)	631 (23.8)	
Middle school	1160 (43.7)	1180 (44.5)	
High school or college	885 (33.3)	838 (31.6)	
Molybdenum (μg/L)	1.24 (0.85–1.70)	1.46 (1.05–1.97)	<0.001

Note: BMI, body mass index; SBP, systolic blood pressure; DBP, diastolic blood pressure; HDL, high density lipoprotein; LDL, low density lipoprotein. Data are presented as *n* (%) for categorical data, means (standard deviations) for parametrically distributed data, or medians (interquartile ranges) for nonparametrically distributed data. ^a^ Missing number was 17, ^b^ Missing number was 28, ^c^ Missing number was 50, ^d^ Missing number was 65, ^e^ Missing number was 51.

**Table 2:** Association of plasma molybdenum concentrations with MetS and its components.

Variables	Quartiles of Plasma Molybdenum Concentrations (μg/L)				Per SD of Log–Transformed Molybdenum	*p*_Trend_
	Q1 (≤1.05)	Q2 (1.05–1.46)	Q3 (1.46–1.97)	Q4 (≥1.97)		
MetS						
No. of cases/controls	1018/669	678/670	555/670	427/669		
Crude OR	1.00	0.66 (0.57–0.76)	0.52 (0.45–0.61)	0.40 (0.34–0.48)	0.71 (0.67–0.76)	<0.001
Model 1 ^a^	1.00	0.67 (0.58–0.79)	0.55 (0.47–0.65)	0.43 (0.36–0.51)	0.73 (0.69–0.78)	<0.001
Model 2 ^b^	1.00	0.70 (0.59–0.82)	0.58 (0.49–0.69)	0.47 (0.39–0.57)	0.76 (0.71–0.82)	<0.001
Model 3 ^c^	1.00	0.71 (0.59–0.84)	0.56 (0.46–0.68)	0.47 (0.39–0.58)	0.77 (0.71–0.83)	<0.001
Abdominal obesity						
No. of cases/controls	1108/569	867/473	721/501	624/471		
Crude OR	1.00	0.94 (0.81–1.10)	0.74 (0.64–0.86)	0.68 (0.58–0.80)	0.88 (0.84–0.94)	<0.001
Model 1 ^a^	1.00	0.86 (0.73–1.01)	0.69 (0.59–0.81)	0.66 (0.56–0.77)	0.87 (0.82–0.92)	<0.001
Model 2 ^b^	1.00	0.77 (0.65–0.91)	0.56 (0.48–0.67)	0.51 (0.43–0.61)	0.77 (0.72–0.82)	<0.001
Model 3 ^c^	1.00	0.80 (0.67–0.95)	0.57 (0.48–0.68)	0.53 (0.43–0.64)	0.79 (0.74–0.85)	<0.001
Hypertriglyceridemia						
No. of cases/controls	872/815	587/760	485/738	420/676		
Crude OR	1.00	0.72 (0.63–0.83)	0.61 (0.53–0.71)	0.58 (0.50–0.68)	0.81 (0.77–0.86)	<0.001
Model 1 ^a^	1.00	0.74 (0.64–0.85)	0.64 (0.55–0.75)	0.60 (0.51–0.70)	0.82 (0.78–0.87)	<0.001
Model 2 ^b^	1.00	0.74 (0.63–0.86)	0.66 (0.56–0.77)	0.61 (0.52–0.72)	0.82 (0.78–0.88)	<0.001
Model 3 ^c^	1.00	0.76 (0.65–0.88)	0.67 (0.56–0.79)	0.64 (0.53–0.76)	0.84 (0.79–0.90)	<0.001
Low HDL–C						
No. of cases/controls	591/1096	481/867	389/835	318/777		
Crude OR	1.00	1.03 (0.89–1.20)	0.86 (0.74–1.01)	0.76 (0.64–0.90)	0.90 (0.85–0.96)	<0.001
Model 1 ^a^	1.00	0.98 (0.84–1.14)	0.87 (0.74–1.03)	0.78 (0.65–0.92)	0.91 (0.85–0.96)	<0.001
Model 2 ^b^	1.00	0.92 (0.79–1.09)	0.79 (0.67–0.94)	0.70 (0.58–0.84)	0.86 (0.81–0.92)	<0.001
Model 3 ^c^	1.00	0.89 (0.75–1.06)	0.77 (0.64–0.92)	0.68 (0.56–0.82)	0.85 (0.79–0.91)	<0.001
High blood pressure						
No. of cases/controls	1127/488	822/501	726/485	644/438		
Crude OR	1.00	0.71 (0.61–0.83)	0.65 (0.55–0.76)	0.64 (0.54–0.75)	0.85 (0.80–0.90)	<0.001
Model 1 ^a^	1.00	0.75 (0.63–0.88)	0.66 (0.56–0.78)	0.63 (0.53–0.75)	0.85 (0.80–0.91)	<0.001
Model 2 ^b^	1.00	0.80 (0.68–0.95)	0.74 (0.62–0.88)	0.75 (0.62–0.90)	0.93 (0.87–1.00)	<0.001
Model 3 ^c^	1.00	0.80 (0.67–0.95)	0.73 (0.61–0.88)	0.71 (0.58–0.86)	0.92 (0.86–0.99)	0.001
Hyperglycemia						
No. of cases/controls	840/845	565/783	495/730	436/659		
Crude OR	1.00	0.73 (0.63–0.84)	0.68 (0.59–0.79)	0.67 (0.57–0.78)	0.82 (0.77–0.86)	<0.001
Model 1 ^a^	1.00	0.74 (0.64–0.86)	0.68 (0.58–0.79)	0.64 (0.55–0.75)	0.81 (0.76–0.85)	<0.001
Model 2 ^b^	1.00	0.89 (0.76–1.04)	0.84 (0.71–0.99)	0.86 (0.72–1.02)	0.92 (0.87–0.98)	<0.001
Model 3 ^c^	1.00	0.90 (0.76–1.06)	0.82 (0.68–0.97)	0.84 (0.69–1.02)	0.92 (0.86–0.99)	0.055

^a^ Model 1: adjusted for sex, age (≤40, 40–49, 50–59, ≥60), and body mass index (≤18, 18–23.9, 24–27.9, ≥28). ^b^ Model 2: adjusted for model 1 plus smoking (current, former, and never), drinking (current, former, and never), physical activity (yes or no), an education level (none or elementary school, middle school, and high school or college), family history of diabetes (yes or no), and survey center (Ezhou and Shenzhen). ^c^ Model 3: adjusted for model 2 plus multiple essential elements (plasma calcium, magnesium, iron, copper, chromium, selenium, and cobalt, all in quartiles).

**Table 3:** Adjusted ORs (95% CIs) for plasma molybdenum concentrations associated with MetS in subgroups.

Subgroup	%	Quartiles of Plasma Molybdenum Concentrations				Per SD of Log-Transformed Molybdenum	*p*_Interaction_
		Q1 (≤1.05)	Q2 (1.05–1.46)	Q3 (1.46–1.97)	Q4 (≥1.97)		
Age							0.18
≤50	35.3	1.00	0.93 (0.71–1.22)	0.66 (0.50–0.88)	0.44 (0.33–0.60)	0.69 (0.62–0.77)	
>50	64.7	1.00	0.61 (0.49–0.74)	0.54 (0.44–0.68)	0.49 (0.38–0.61)	0.77 (0.71–0.84)	
Sex							0.002
Men	55.2	1.00	0.57 (0.46–0.72)	0.53 (0.42–0.66)	0.44 (0.34–0.56)	0.74 (0.68–0.80)	
Women	44.8	1.00	0.94 (0.74–1.18)	0.71 (0.55–0.91)	0.58 (0.44–0.76)	0.80 (0.72–0.88)	
BMI							<0.001
≤24	32.7	1.00	0.39 (0.28–0.55)	0.19 (0.13–0.26)	0.12 (0.08–0.18)	0.43 (0.37–0.50)	
>24	67.3	1.00	0.85 (0.71–1.02)	0.87 (0.71–1.06)	0.79 (0.64–0.98)	0.92 (0.85–0.99)	
Current Smoking							0.067
No	77.1	1.00	0.76 (0.63–0.91)	0.65 (0.54–0.79)	0.52 (0.42–0.64)	0.76 (0.71–0.82)	
Yes	22.9	1.00	0.57 (0.40–0.81)	0.41 (0.28–0.59)	0.39 (0.26–0.58)	0.73 (0.64–0.84)	
Current Drinking							0.017
No	73.6	1.00	0.80 (0.66–0.96)	0.71 (0.59–0.86)	0.57 (0.46–0.71)	0.81 (0.75–0.87)	
Yes	26.4	1.00	0.56 (0.41–0.78)	0.37 (0.26–0.51)	0.32 (0.23–0.46)	0.65 (0.57–0.74)	
Physical activity							0.603
No	43.6	1.00	0.65 (0.50–0.83)	0.52 (0.40–0.67)	0.45 (0.35–0.60)	0.72 (0.65–0.79)	
Yes	56.4	1.00	0.80 (0.65–0.98)	0.67 (0.54–0.84)	0.51 (0.40–0.66)	0.79 (0.72–0.86)	
Family history of diabetes							0.427
No	88.8	1.00	0.70 (0.59–0.83)	0.59 (0.49–0.70)	0.48 (0.40–0.58)	0.76 (0.71–0.82)	
Yes	11.2	1.00	0.86 (0.53–1.40)	0.68 (0.40–1.14)	0.47 (0.27–0.82)	0.74 (0.61–0.89)	
Survey center							0.467
Ezhou	53.8	1.00	0.60 (0.48–0.75)	0.50 (0.39–0.63)	0.41 (0.31–0.54)	0.71 (0.65–0.78)	
Shenzhen	46.2	1.00	0.85 (0.67–1.07)	0.67 (0.52–0.85)	0.54 (0.42–0.71)	0.81 (0.74–0.88)	

Results were adjusted for sex, age (≤40, 40–49, 50–59, ≥60), body mass index (≤18, 18–23.9, 24–27.9, ≥28), smoking (current, former, and never), drinking (current, former, and never), physical activity (yes or no), an education level (none or elementary school, middle school, and high school or college), family history of diabetes (yes or no), survey center (Ezhou and Shenzhen), and multiple essential elements (plasma calcium, magnesium, iron, copper, chromium, selenium, and cobalt, all in quartiles).
